# Watt-level passively Q-switched heavily Er^3+^-doped ZBLAN fiber laser with a semiconductor saturable absorber mirror

**DOI:** 10.1038/srep26659

**Published:** 2016-05-26

**Authors:** Yanlong Shen, Yishan Wang, Kunpeng Luan, Ke Huang, Mengmeng Tao, Hongwei Chen, Aiping Yi, Guobin Feng, Jinhai Si

**Affiliations:** 1 State Key Laboratory of Transient Optics and Photonics, Xi’an Institute of Optics and Precision Mechanics, Chinese Academy of Sciences, Xi’an Shaanxi 710119, China; 2 Shaanxi Key Laboratory of Photonics Technology for Information, School of Electronics and Information Engineering, Xi’an Jiaotong University, Xi’an, Shaanxi 710049, China; 3 State Key Laboratory of Laser Interaction with Matter, Northwest Institute of Nuclear Technology, Xi’an Shaanxi 710024, China; 4 University of Chinese Academy of Sciences, Beijing, 100049, China; 5 Collaborative Innovation Center of Extreme Optics, Shanxi University, Taiyuan, Shanxi, 030006, China

## Abstract

A diode-cladding pumped mid-infrared passively Q-switched Er^3+^-doped ZBLAN fiber laser with an average output power of watt-level based on a semiconductor saturable absorber mirror (SESAM) is demonstrated. Stable pulse train was produced at a slope efficiency of 17.8% with respect to launched pump power. The maximum average power of 1.01 W at a repetition rate of 146.3 kHz was achieved with a corresponding pulse energy of 6.9 μJ, from which the maximum peak power was calculated to be 21.9 W. To the best of our knowledge, the average power and the peak power are the highest in 3 μm region passively Q-switched fiber lasers. The influence of gain fiber length on the operation regime of the fiber laser has been investigated in detail.

Recently, rare-earth ions doped fluoride fiber lasers emitting near 3 μm waveband have received much interest owing to the combination of their potential applications in laser medicine, spectroscopy, infrared countermeasures and mid-IR laser pumping^1^,^2^,^3^,^4^, and their inherent advantages of fiber lasers, including high conversion efficiency, excellent beam quality, compactness and tunable output^5^, etc. Thanks to commercially available high power laser diode as pumping sources, significant progresses of continuous wave (CW) ZBLAN fiber lasers at 3 μm have been achieved over the past decade^1^,^2^,^3^,^4^,^6^,^7^,^8^,^9^,^10^,^11^, and the maximum recorded power in excess of 20 W was obtained^8^,^9^. However, some special applications, such as, material processing, micro surgery and nonlinear optical process, require a high pulse energy laser source, and thus Q-switched operation of ZBLAN fiber lasers is highly desired and thereby prior to CW and mode-locking operation^12^. A number of actively Q-switched Er^3+^-doped and Ho^3+^-doped(Ho^3+^/Pr^3+^-co-doped) fluoride fiber lasers with emission at 2.8 μm and 2.9 μm respectively have been demonstrated based on an acousto-optic modulator^13^,^14^,^15^,^16^,^17^,^18^. The first actively Q-switched fluoride fiber laser at 3 μm region with an acousto-optic modulator was reported in early 1994^13^. With the development of laser diode (LD), the output power was scaled up significantly in the past years. An actively Q-switched Er^3+^-doped ZBLAN fiber laser at 2.8 μm with a pulse energy of 100 μJ, average power of 12 W, and peak power of 0.9 kW, was obtained in 2011^14^. Subsequently, an actively Q-switched Ho^3+^/Pr^3+^ co-doped ZBLAN fiber laser at 2.87 μm producing 78 ns pulses with a peak power of 77 W at a repetition rate up to 300 kHz was reported^15^, and the shortest Q-switched pulse at 3 μm with a pulse width of 33 ns and peak power of 576 W was obtained at a relatively low repetition rate^16^. Meanwhile, a dual-wavelength actively Q-switched singly Ho^3+^-doped cascade fiber laser with a pulse energy of 29 μJ, and a pulse width of 380 ns at mid-infrared emission of 3.005 μm was demonstrated^17^.

Compared to bulky and complicated active Q-switching schemes, passive Q-switching has advantages of simplicity, compactness and especially no need of additional electric device for Q-switches^19^. In the past decade, many demonstrations of passively Q-switched Er^3+^-doped and Ho^3+^ doped ZBLAN fiber laser were reported by employing various saturable absorbers, including InAs epilayers^20^, Fe^2+^:ZnSe crystal^19^,^21^, graphene^19^,^22^ and SESAM^12^,^23^,^24^. Zhu *et al.* demonstrated a passively Q-switched Er^3+^-doped and Ho^3+^-doped ZBLAN fiber lasers with pulse widths of 370 ns and 800 ns, pulse energies of 2.0 μJ and 460 nJ at repetition rates of 161 kHz and 105 kHz by using a Fe^2+^:ZnSe crystal, respectively^19^,^21^. By replacing the Fe^2+^:ZnSe crystal with graphene, they also achieved passively Q-switched pulses through Er^3+^-doped and Ho^3+^-doped ZBLAN fibers. Moreover, Li *et al.* reported a passively switched dual wavelength Ho^3+^-doped fluoride fiber laser with a pulse energy of 6.16 μJ, a pulse width of 0.73 μs and a repetition rate of 51.1 kHz with a calculated peak power of 8.4 W at 3 μm not long ago by using a SESAM^24^, which possessed the maximum peak power in 3 μm waveband passively Q-switched fiber lasers up to now. With the emergence of new materials used as saturable absorbers, topological insulator and black phosphorus have been demonstrated to have the capability of generating mid-infrared laser pulses very recently^25^,^26^. During these passively Q-switched demonstrations, the maximum average power and the maximum peak power were 629.2 mW and 8.4 W, respectively.

In this paper, we report a watt-level passively Q-switched fluoride fiber laser at 3 μm waveband using a SESAM. A pulse energy of 6.9 μJ and a pulse width of 315 ns at a repetition rate of 146.5 kHz are achieved with a peak power of exceeding 21 W by employing a heavily Er^3+^-doped ZBLAN fiber as short as 0.9 m. The average power and the peak power, to our knowledge, are current recorded in passively Q-switched fiber lasers at mid-infrared region.

## Results

**Figure 1:**
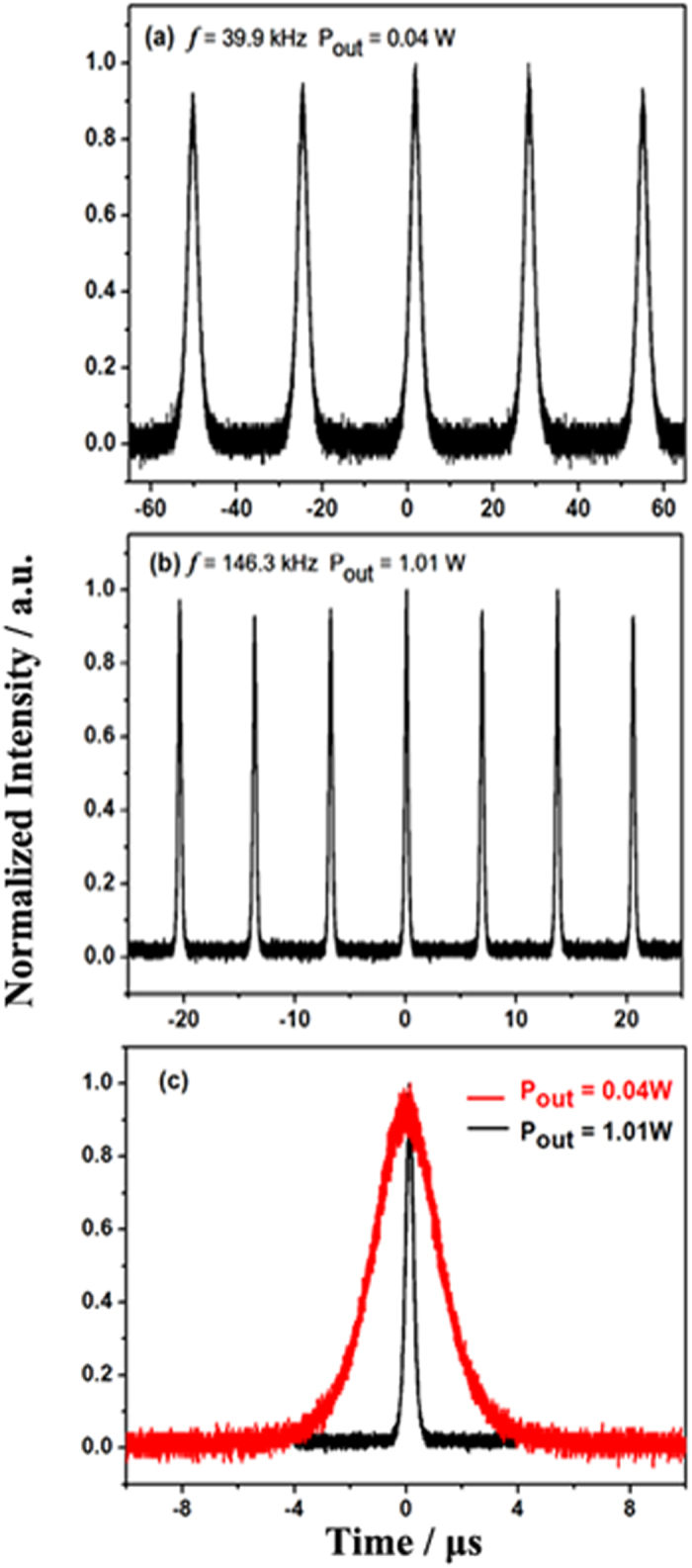
Typical Q-switched pulse trains at the output powers of (**a**) 0.04 W and (**b**) 1.01 W, followed with (**c**) comparison of their individual pulse envelopes of the longest and shortest stable pulses.

As increasing the pump power, the fiber laser went through three stages, e.g., CW regime, unstable Q-switching and stable Q-switching, which were analogous to ref. 12. The threshold of launched pump power for CW operation was 0.52 W. The laser started to work in Q-switching when the launched pump power was 0.59 W, whereas the pulse trains were unstable. Stable, passively Q-switched pulses took place at a pump power of 0.84 W, with a repetition rate of 39.9 kHz, as shown in Fig. 1(a,c). As we increased the launched pump power from 0.84 W to the maximum power of 6.27 W, the average output power was promoted from 0.04 W to 1.01 W, and the pulse repetition rate of the Q-switched Er^3+^-doped ZBLAN fiber laser was changed to 146.3 kHz with a much narrower pulse width, as shown in Fig. 1(b,c).

**Figure 2:**
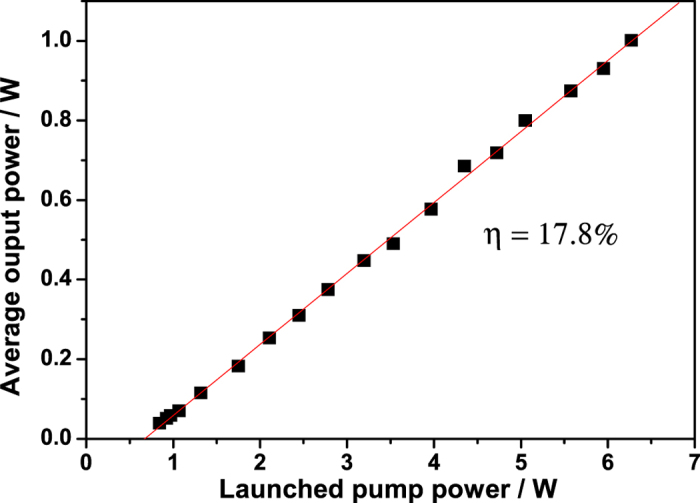
Average output power varies with launched pump power.

Average output power versus launched pump power is plotted in Fig. 2. The maximum output power obtained was greater than 1 W with a slope efficiency of 17.8% with respect to launched pump power. The slop efficiency of was nearly closed to ref. 17 and slightly lower than CW regime in our present experiment due to intracavity loss introduced by the SESAM. The linearity of the curve verifies the output power could be scaled up as well.

**Figure 3:**
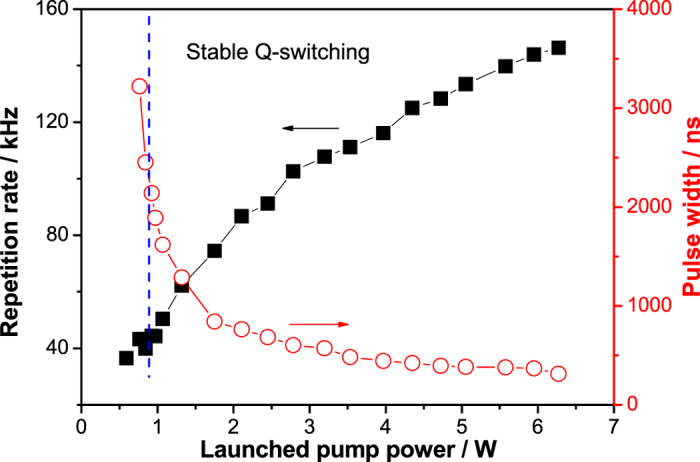
Measured repetition rate (black line with squares) and pulse width (red line with circles) as a function of launched pump power.

**Figure 4:**
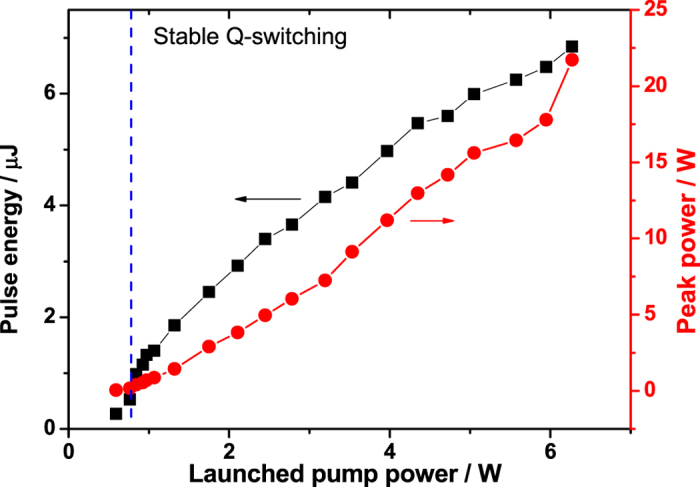
Calculated pulse energy (black line with squares) and peak power (red line with dots) versus launched pump power.

Figure 3 shows the measured repetition rate and pulse width as a function of launched pump power. As expected and observed in typical passively Q-switched fiber lasers^19^, the repetition rate increased almost linearly with the increased launched pump power from 39.9 kHz to 146.3 kHz, whereas the pulse width decreased from 2.75 μs to as short as 315 ns, which would be further shortened by reducing the laser cavity length^14^. Combining the average output power gave the calculated pulse energy and peak power, as shown in Fig. 4. The pulse energy and average output power of the stable Q-switched fiber laser both increased monotonically with launched pump power. The maximum average power of 1.01 W and corresponding pulse energy of 6.9 μJ was obtained at the maximum launched pump power of 6.27 W, yielding the maximum peak power of 21.9 W, which was the highest peak power reported to date, to our knowledge, in the passively Q-switched 3 μm region fiber lasers.

**Figure 5:**
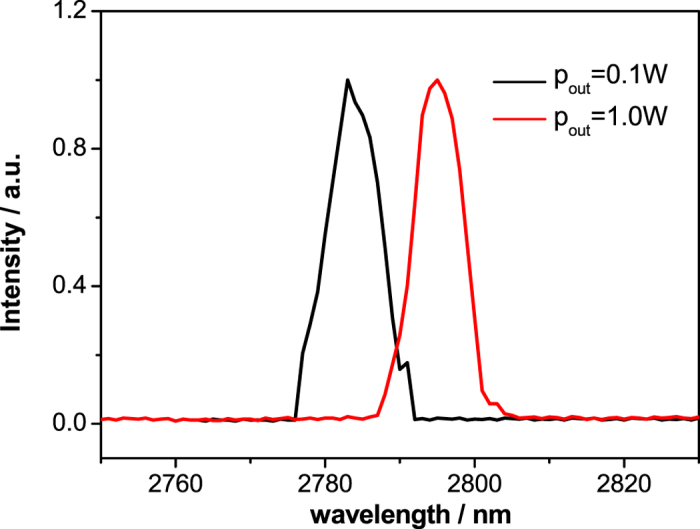
Output spectra at different output powers.

The measured output spectra at different output powers are shown in Fig. 5. The central wavelength of 2783.0 nm at lower output power shifted to 2794.9 nm at the maximum output power, with almost the same bandwidth (FWHM) of ~8 nm.

## Discussion

As increasing the pump power, the temperature of the heat sink of the SESAM was rising, mainly due to the residual pump power deposited in the SESAM if active cooling was not used, which could result in instability of the pulsed output. By employing air flow with a fan for actively cooling the heat sink, the Q-switched pulse output was stable at the maximum output power with pulse-to-pulse amplitude stability of less than 5%. When we stopped the cooling for seconds, the heat sink of the SESAM was hot and the output average power fluctuated, which might be caused by thermal effect of the SESAM. No damage to the fiber ends and the SESAM was observed in the course of the present experiments, even when it was operating at the maximal power. The average power of the fiber laser at stable pulsed output could be scaled up by preventing the residual pump power injecting into the SESAM through a high reflection (HR) coated film at pump wavelength in the surface of the SESAM or a dichroic mirror(HR at pump wavelength) between the active fiber end and the SESAM. The linearity of the plot in Fig. 2 and no observed saturation also indicated the average power could be promoted by increasing the pump power.

The shift of the central wavelength shown in Fig. 5 is also named red-shift, which is very common in lasers without wavelength selecting elements in the cavity when the pump power increasing^2^, due to the fact that the more the pump power, the higher the temperature of the active medium, and the lower the initial Stark level of upper laser level^12^. The stability of output spectrum would be improved greatly and the bandwidth could be narrowed significantly by using a bulk grating or a fiber Bragg grating in the laser cavity^2^.

In comparison with the SESAM-based passively switched demonstration including passively Q-switching and mode-locking, only Q-switched operation^12^, i.e., pure passive Q-switching^27^, was observed in our case as well. According to the passively switched theory with a SESAM, we could estimate the length of active fiber with which the laser operated in pure Q-switched regime at watt-level without mode-locking using the following condition^12^,^27^:


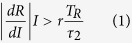


where *R* and *I* represent the SESAM reflectivity and laser intensity in the SESAM, respectively. *r* represent the pump to threshold ratio, which is about 12.1 in our case. *τ*_2_ is upper laser state lifetime, which is 6.9 ms^2^. The physical interpretation of (1) is as follows: The left side of Eq. 1 determines absorber reflectivity, i.e., the reduction in losses per cavity round-trip due to the bleaching in the saturable absorber. This loss reduction will increase the intensity inside the laser cavity. The right side of (1) determines how much the gain per round-trip saturates. For a certain parameter r, the gain is supposed to be constant. If the loss reduced enough, the intensity continues to increase as the absorber is bleached, delivering a giant pulse, and thus resulting in Q-switching^27^. *T*_*R*_ is cavity round trip time, which is expressed as:


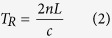


where *n* and *c* are the SESAM reflectivity and the speed of light in vacuum, respectively. *L* is the length of laser cavity, which is equal to the length of the active fiber in our case.

Substituting (2) to (1), yields:


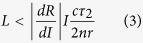


Assuming the average output power of 1 W, combining the thickness of the SESAM and parameters of the fiber core and core NA, we could roughly calculate the laser intensity in the SESAM which is around 3.41 × 10^8^ μW/cm^2^. Considering the information provided by the manufacturer, the deduced 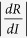 is about 6.0 × 10^−14^(μW/cm^2^)^−1^. Taking all the values into (3) gives L < 1.17 m. That explains why pure passive Q-switching was observed in above experiment. Moreover, we also performed an analogous experiment with all the same conditions except just replacing the active fiber with a longer one, whose length is around 3.3 m. The longer fiber had all same parameters with the shorter one, including core diameter, core NA, concentration, etc. When the output power exceeded 1 W (while the parameter r did not change much), the laser was in Q-switched mode-locking regime.

As estimated in passively Q-switched fiber lasers with a saturable absorber^12^, pulse width was of the order of *T*_*R*_/Δ*R*, where *T*_*R*_ was the time of round-trip in the cavity, and Δ*R* was the modulation depth of the SESAM. The theoretical limit of pulse width was of 250 ns in our case, which was closed to the value we achieved above. Moreover, the pulse width could be further reduced only by replacing the SESAM with a greater modulation depth one^28^.

## Methods

**Figure 6:**
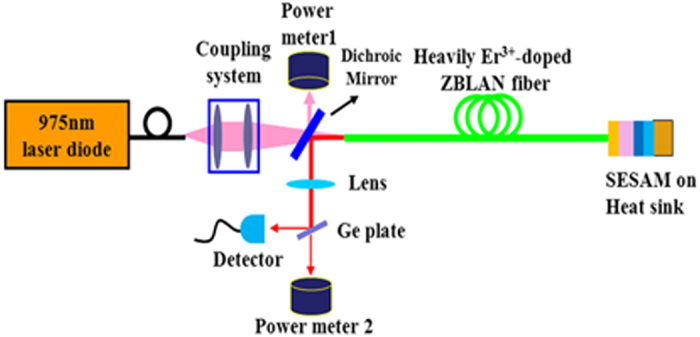
Schematic layout of experimental setup of SESAM-based passively Q-switched Er^3+^-doped ZBLAN fiber laser.

The experimental setup for the SESAM-based passively Q-switched Er^3+^-doped ZBLAN fiber laser is schematically illustrated in Fig. 6. A commercially available fiber-coupled laser diode, centered at around 975 nm, was used as a pumping source. The pigtail fiber of the laser diode had a core diameter of 105 μm and a numerical aperture (NA) of 0.22. The active fiber (fabricated by FiberLabs, Inc.) was a piece of heavily Er^3+^-doped ZBLAN double-clad fiber with a length of about 0.9 m. The specifications of the active fiber were as follows: core diameter, 33 μm; core NA, 0.12; ErF_3_ concentration, 6 mol%; inner cladding diameter, 330 μm; inner cladding NA, 0.55. Both ends of the fibers were held by fiber chuck holders with a U shaped groove heat sink. The pump absorption efficiency of this active fiber was measured to be around 80%. The 975 nm LD pump beam was coupled into the inner cladding of the active fiber by a coupling system composed of a collimator and an aspheric lens. Each side of the lenses was antireflection coated at the pump wavelength. With this pump configuration, the efficiency of the coupling system was measured to be ~80%.

In our laser cavity arrangement, the pumping end of the fiber was carefully cleaved by a cleaver at 0° to enable the pumping end with Fresnel reflection (4%) to work as the laser output port. In front of the pumping end, a dichroic mirror (high reflection ~99% at 2.8 μm, high transmittance >98% at 975 nm) was placed with an incidence angle of 45° to couple out the laser beam. The feedback of the cavity was provided by a piece of reverse designed SESAM (BATOP GmbH). It is worth pointing out that, unlike inserting collimators in cavities^12^,^23^, the SESAM was physically and directly contacted against the rear end of the active fiber to simplify the cavity design and adjustment. Follows were some typical parameters of the SESAM (provided by the manufacturer). The central wavelength of reflectance was 2900 nm with a waveband ranging from 2400 to 3000 nm accompanied with a relaxation time of around 10 ps. The absorbance and modulation depth were 9% and 4%, respectively. While the saturation fluence and damage threshold were 150 μJ/cm^2^ and 2 mJ/cm^2^, respectively.

The launched pump power was synchronously monitored through a fraction (~1%) of the pump beam reflected by the dichroic mirror with a power meter (Gentec, XLP12-3S-H2-D0). A CaF_2_ lens with a focal length of 50 mm was used to collimate the output laser beam. An uncoated Germanium plate of 2 mm thickness (Transmittance ~45% at >2 μm, Transmittance ~0 at <1.8 μm) was located to purify the output and split the output into two parts. One part was measured with a power meter (Gentec, UP19k-50L-H5), and the other was detected with an HgCdTe detector with a rise time of less than 3 ns (Vigo PVM-2TE-10.6-2). The waveforms of the laser signal were displayed with a 1 GHz digital oscilloscope. The output spectrum was measured with a spectrometer (Andor, Shamrock 750).

## Conclusion

In conclusion, we have demonstrated a diode-cladding pumped passively SESAM-based Q-switched mid-infrared Er^3+^-doped ZBLAN fiber laser. Stable pulse train was produced with the maximum average power of 1.01 W, a repetition rate of 146.3 kHz, and a corresponding pulse width of 315 ns at a slope efficiency of 17.8% with respect to launched pump power. The calculated maximum pulse energy of 6.9 μJ and the calculated maximum peak power of 21.9 W were achieved, respectively. This work provided a way to obtain pure passively Q-switched 3 μm waveband fiber laser, since no damage of the SESAM was observed after extended operation at the maximum pump power, higher output power and energy pulses are expected by increasing the pump power.

## Additional Information

**How to cite this article**: Shen, Y. *et al.* Watt-level passively Q-switched heavily Er^3+^-doped ZBLAN fiber laser with a semiconductor saturable absorber mirror. *Sci. Rep.* **6**, 26659; doi: 10.1038/srep26659 (2016).

## References

[b1] Sumiyoshi T. *et al.* High-power continuous-wave 3-and 2-μm cascade Ho^3+^: ZBLAN fiber laser and its medical applications. IEEE J. Sel. Top. Quant. 5, 936–943 (1999).

[b2] Zhu X. & Jain R. 10-W-level diode-pumped compact 2.78 μm ZBLAN fiber laser. Opt. Lett. 32, 26–28 (2007). 17167572 10.1364/ol.32.000026

[b3] Jackson S. D., King T. A. & Pollnau M. Diode-pumped 1.7-W erbium 3-μm fiber laser. Opt. Lett. 24, 1133–1135 (1999). 18073963 10.1364/ol.24.001133

[b4] Srinivasn B., Tafoya J. & Jain R. High-power “Watt-level” CW operation of diode-pumped 2.7 μm fiber lasers using efficient cross-relaxation and energy transfer mechanisms. Opt. Express 4, 490–495 (1999). 19396307 10.1364/oe.4.000490

[b5] Zhu X. & Jain R. Numerical analysis and experimental results of high-power Er/Pr:ZBLAN 2.7 μm fiber lasers with different pumping designs. Appl. Optics. 45, 7118–7125 (2006). 10.1364/ao.45.007118 16946791

[b6] Jackson S. D. Single-transverse-mode 2.5-W holmium-doped fluoride fiber laser operating at 2.86 μm. Opt. Lett. 29, 334–336 (2004). 14971744 10.1364/ol.29.000334

[b7] Zhu X. & Jain R. Compact 2 W wavelength-tunable Er: ZBLAN mid-infrared fiber laser. Opt. Lett. 32, 2381–2383 (2007). 17700792 10.1364/ol.32.002381

[b8] Tokita S., Murakami M., Shimizu S., Hashida M. & Sakabe S. Liquid-cooled 24 W mid-infrared Er:ZBLAN fiber laser. Opt. Lett. 34, 3062–3064 (2009). 19838226 10.1364/OL.34.003062

[b9] Faucher D., Bernier M., Androz G., Caron N. & Vallée R. 20 W passively cooled single-mode all-fiber laser at 2.8 μm. Opt. Lett. 36, 1104–1106 (2011). 21478997 10.1364/OL.36.001104

[b10] Tokita S. *et al.* Stable 10 W Er: ZBLAN fiber laser operating at 2.71-2.88 μm. Opt. Lett. 35, 3943–3945 (2010). 21124573 10.1364/OL.35.003943

[b11] Li J., Hudson D. D. & Jackson S. D. High-power diode-pumped fiber laser operating at 3 μm. Opt. Lett. 36, 3642–3644 (2011). 21931418 10.1364/OL.36.003642

[b12] Li J. *et al.* Semiconductor saturable absorber mirror passively Q-switched 2.97 μm fluoride fiber laser. Laser Phys. Lett. 11, 065102 (2014).

[b13] Frerichs C. & Tauermann T. Q-switched operation of laser diode pumped erbium-doped fuorozirconate fibre laser operating at 2.7 μm. Electron. Lett. 30, 706–707 (1994).

[b14] Tokita S., Murakami M., Shimizu S., Hashida M. & Sakabe S. 12 W Q-switched Er:ZBLAN fiber laser at 2.8 μm. Opt. Lett. 36, 2812–2814 (2011). 21808321 10.1364/OL.36.002812

[b15] Hu T., Hudson D. D. & Jackson S. D. Actively Q-switched 2.9 μm Ho^3+^ Pr ^3+^-doped fluoride fiber laser. Opt. Lett. 37, 2145–2147 (2012). 22660149 10.1364/OL.37.002145

[b16] Hu T., Hudson D. D. & Jackson S. D. High peak power actively Q-switched Ho^3+^, Pr ^3+^-co-doped fluoride fiber laser. Electron. Lett. 49, 1–2 (2013).

[b17] Li J., Hu T. & Jackson S. D. Dual wavelength Q-switched cascade laser. Opt. Lett. 37, 2208–2210 (2012). 22739857 10.1364/OL.37.002208

[b18] Li J., Yang Y., Hudson D. D., Liu Y. & Jackson S. D. A tunable Q-switched Ho^3+^-doped fluoride fiber laser. Laser Phys. Lett. 10, 045107 (2013).

[b19] Zhu G., Zhu X., Balakrishnan K., Norwood R. A. & Peyghambarian N. Fe^2+^: ZnSe and graphene Q-switched singly Ho^3+^-doped ZBLAN fiber lasers at 3 μm. Opt. Mater. Express 3, 1365–1377 (2013).

[b20] Frerichs C. & Unrau U. B. Passive Q-switching and mode-locking of erbium-doped fluoride fiber lasers at 2.7 μm. Opt. Fiber Technol. 2, 358–366 (1996).

[b21] Wei C., Zhu X., Norwood R. A. & Peyghambarian N. Passively Q-Switched 2.8 μm Nanosecond Fiber Laser. IEEE Photonic. Technol. L. 24, 1741–1744 (2012).

[b22] Wei C. *et al.* Graphene Q-switched 2.78 μm Er^3+^-doped fluoride fiber laser. Opt. Lett. 38, 3233–3236 (2013). 23988922 10.1364/OL.38.003233

[b23] Li J., Hudson D. D., Liu Y. & Jackson S. D. Efficient 2.87 μm fiber laser passively switched using a semiconductor saturable absorber mirror. Opt. Lett. 37, 3747–3749 (2012). 23041846 10.1364/ol.37.003747

[b24] Li J. *et al.* Mid-infrared passively switched pulsed dual wavelength Ho^3+^-doped fluoride fiber laser at 3 μm and 2 μm. Sci. Rep. 5, 10770 (2015) 26041105 10.1038/srep10770 PMC4455194

[b25] Li J. *et al.* 3-μm mid-infrared pulse generation using topological insulator as the saturable absorber. Opt. Lett. 40, 3659–3662 (2015). 26258382 10.1364/OL.40.003659

[b26] Qin Z. *et al.* Black phosphorus as saturable absorber for the Q-switched Er:ZBLAN fiber laser at 2.8 μm. Opt. Express. 23, 24713–24718 (2015). 26406672 10.1364/OE.23.024713

[b27] Keller U. *et al.* Semiconductor saturable absorber mirrors (SESAM’s) for femtosecond to nanosecond pulse generation in solid-state lasers. IEEE J. Sel. Top. Quant. 2, 435–453 (1996).

[b28] Hakulinen T. & Okhotnikov O. G. 8 ns fiber laser Q switched by the resonant saturable absorber mirror. Opt. Lett. 32, 2677–2679 (2007). 17873932 10.1364/ol.32.002677

